# Giant Paratesticular Myxoid Liposarcoma: A Case Report of a Rare Entity

**DOI:** 10.7759/cureus.56859

**Published:** 2024-03-25

**Authors:** Vettrivizhi Sampath Arutperumselvi, Karthik Krishna Ramakrishnan, Vinoth Pandian, Yuvaraj Muralidharan, Chakradhar Ravipati

**Affiliations:** 1 Department of Radiology, Saveetha Medical College and Hospital, Saveetha Institute of Medical and Technical Sciences, Saveetha University, Tamil Nadu, Chennai, IND

**Keywords:** testicular malignancy, orchidectomy, scrotal swelling, adipose tissue tumour, paratesticular myxoid liposarcoma

## Abstract

Paratesticular myxoid liposarcoma is an exceedingly rare malignancy originating from the spermatic cord or paratesticular tissues. We report a unique case of a 75-year-old male patient who presented with a painless scrotal swelling that had been growing for four years. Imaging investigations, including ultrasonography (USG) and contrast-enhanced computerized tomography (CECT), revealed characteristics consistent with paratesticular myxoid liposarcoma. The orchidectomy specimen confirmed a grade 2 right paratesticular myxoid liposarcoma. Despite its rarity, clinicians must consider this tumor in the differential diagnosis of painless scrotal swellings. Accurate diagnosis and comprehensive management, encompassing surgical resection with wide margins and potential adjuvant therapies, are pivotal. This case underlines the importance of collaborative research and long-term follow-up in understanding and managing paratesticular myxoid liposarcomas.

## Introduction

Paratesticular myxoid liposarcoma (PTML) is a rare malignant soft tissue tumor originating from adipose tissue in the paratesticular region. It is characterized by a myxoid matrix with scattered lipoblasts, making it distinct from other liposarcoma subtypes. While myxoid liposarcomas can occur in various anatomical sites, paratesticular involvement is particularly unusual and presents diagnostic and therapeutic challenges due to its anatomical proximity to vital structures. The peak incidence of paratesticular sarcomas is seen in men in their 60s. Usually, it presents with a painful or painless scrotal mass or swelling, occasionally accompanied by a hydrocele [1]. Paratesticular liposarcoma is an infrequent tumor characterized by a growing painless inguinal or scrotal mass. Only about 250 cases have been reported as of yet in literature; however, there are a few cases regarding giant paratesticular liposarcoma measuring over 10 cm. Myxoid liposarcoma is the most common histological subtype, and giant liposarcomas are those measuring over 10 cm. It is commonly misdiagnosed before histopathological evaluation leading to local recurrence due to inaccurate diagnosis and treatment. Liposarcoma can originate from the tissue of the cord, which can be an extension of retroperitoneal fat, or it can result from the malignant transformation of a preexisting lipoma [2].

## Case presentation

A 75-year-old male from a low socioeconomic background presented with a painless right scrotal swelling that had gradually grown over four years. He expressed concern about the persistent enlargement, denying any history of trauma, fever, or weight loss. Clinical examination revealed a firm, non-tender mass in the right scrotum, measuring approximately 12 cm in maximum diameter. This clinical observation highlighted the prominence and significance of the mass as the primary focus of concern. The lack of additional positive findings underscored the discreet nature of the manifestations, emphasizing the need for thorough diagnostic investigations to unravel the nature and implications of the scrotal mass. Financial constraints limited previous healthcare access, and the patient lacked a significant medical history. His family history was unremarkable. Ultrasonography (USG) and subsequent contrast-enhanced computerized tomography (CECT) were pursued to investigate the swelling. The imaging findings guided the decision for a high orchidectomy. The subsequent diagnosis of a grade 2 right paratesticular myxoid liposarcoma (PTML) brought forth challenges in the context of limited resources, emphasizing the need for accessible and inclusive healthcare solutions.

Investigations

**Figure 1:**
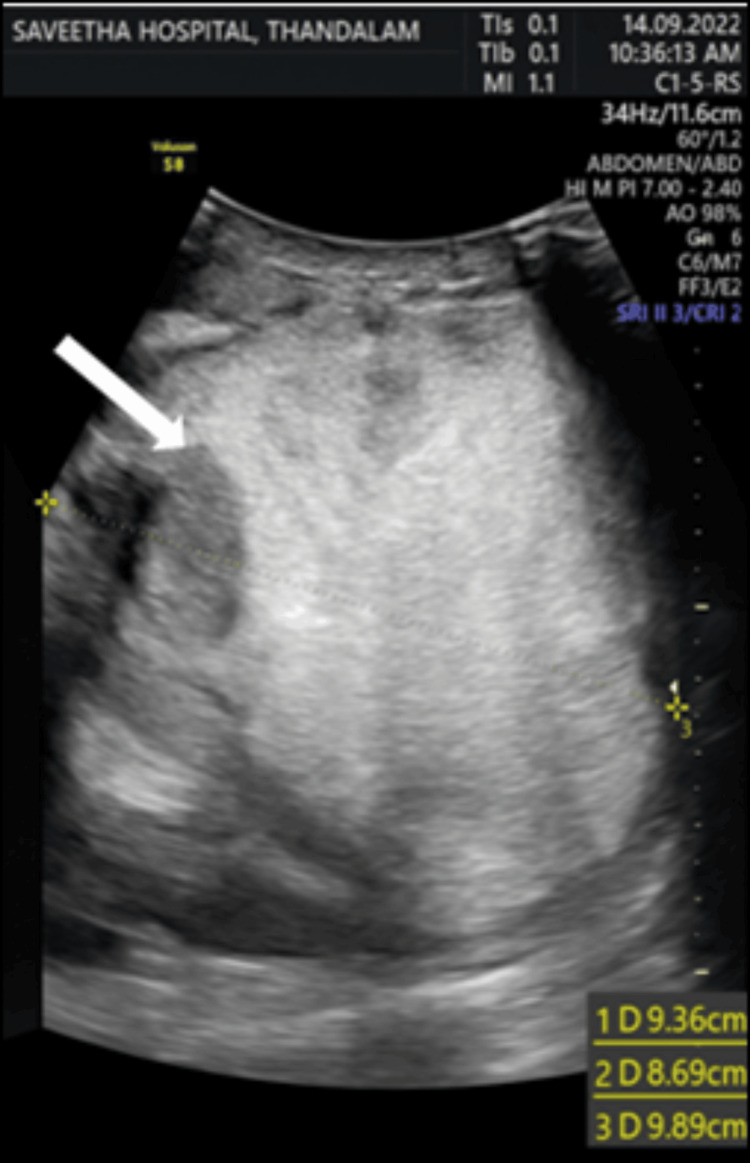
Scrotal ultrasound showed a large heteroechoic solid mass (white arrow) measuring approximately 9.9 x 9.4 x 8.7 cm, with hyperechoic areas seen expanding the right hemi-scrotal sac. Both testicles were seen separately; however, the testicular origin of the mass lesion could not be confirmed, necessitating cross-sectional imaging.

The diagnostic journey incorporated a comprehensive assessment, including laboratory examinations. Hemogram, urinalysis, stool routine, ESR, β-human chorionic gonadotropin, α-fetoprotein, mycobacterium tuberculosis antibody Ig-G, liver and kidney function tests, and chest x-ray were conducted. All these investigations yielded no specific abnormalities, contributing to the challenge of diagnosing the scrotal mass. Despite the absence of abnormalities in these baseline assessments, imaging modalities, including USG and CECT were performed to characterize the scrotal mass. USG revealed a large heteroechoic solid mass (~9.9 x 9.4 x 8.7 cm) with hyperechoic areas seen expanding the right hemiscrotal sac (Figure 1). 

**Figure 2:**
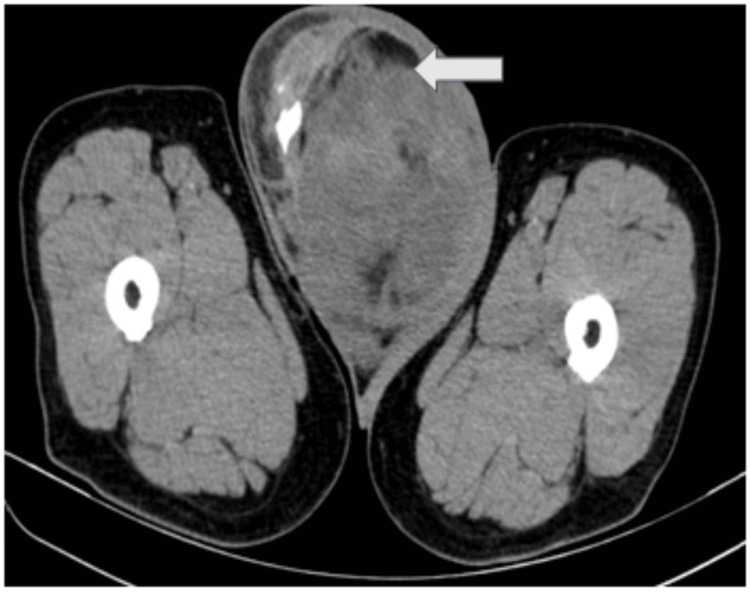
NECT abdomen axial section showing a large mild heterogeneous mixed attenuating extratesticular lesion measuring ~12.7 x 10.9 x 9.3 cm with internal soft tissue and fat attenuating areas and a few peripheral punctate & chalky calcifications seen involving and expanding the right hemi-scrotum (white arrow). NECT - Non-enhanced computerized tomography

**Figure 3:**
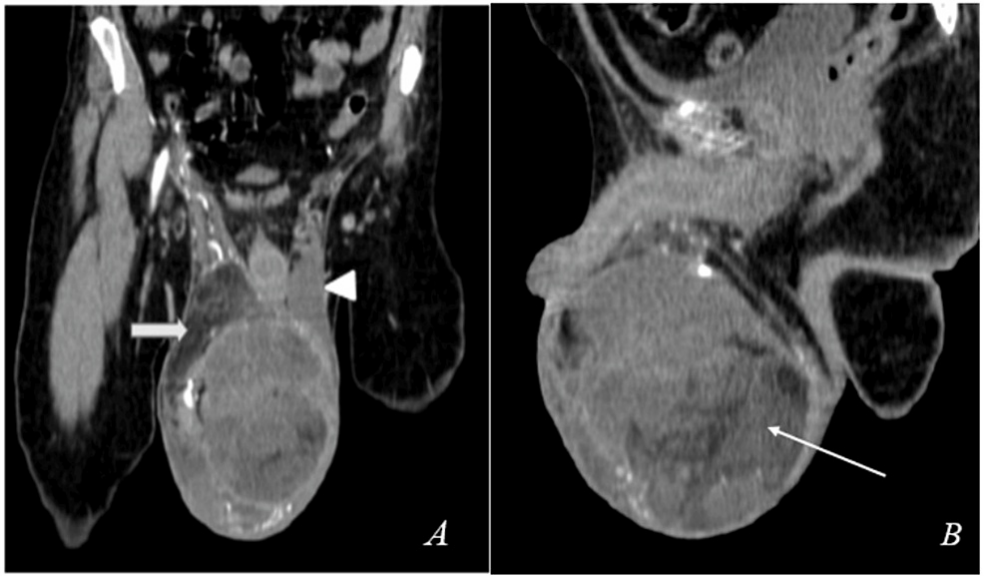
CECT abdomen coronal section (A) and sagittal section (B) showing a large mild heterogeneously enhancing mixed attenuating extratesticular lesion measuring approximately 12.7 x 10.9 x 9.3 cm with internal soft tissue and fat attenuating areas and a few peripheral punctate & chalky calcifications seen involving and expanding the right hemi-scrotum with lipomatous part of the tumour of right spermatic cord (white arrow in image A, white arrow in image B). The lesion is seen causing inferior and peripheral displacement of the right testis and significant compression and cranial displacement of the left hemi-scrotum and testis (white arrow head). CECT - Contrast-enhanced computed tomography

CECT of the abdomen and pelvis revealed a large heterogeneously enhancing mixed attenuating extratesticular lesion (~ 12.7 x 10.9 x 9.3 cm) with internal soft tissue and fat attenuating areas and peripheral punctate & chalky calcifications were evident in the right hemi-scrotum, along with lipomatous hypertrophy of right paratesticular tissue (spermatic cord). This mass lesion was causing inferior and peripheral displacement of the right testis and significant compression and cranial displacement of the left hemi-scrotum and testis, as can be seen in Figures 2, 3A, 3B.

**Figure 4:**
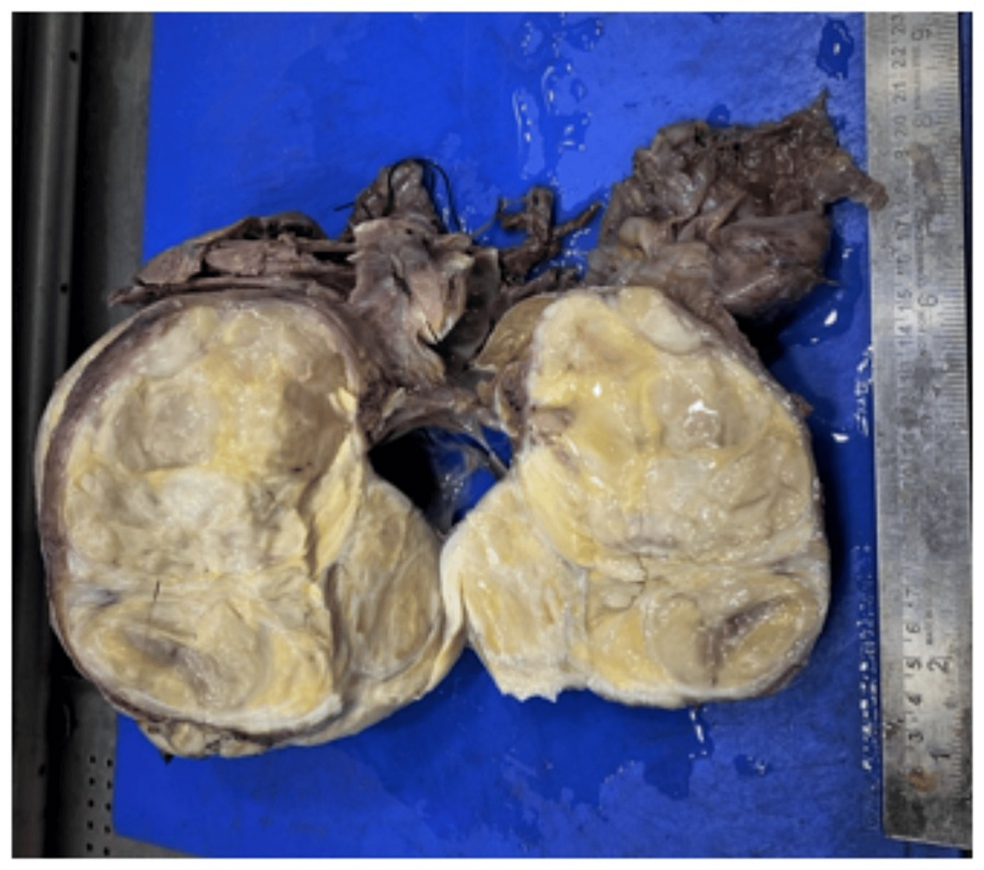
Gross image of the lesion showed solid lobulated paratesticular mass with yellowish mucoid areas on the right side.

A postoperative gross specimen of the lesion was obtained, and it showed a solid, lobulated para-testicular mass with yellowish mucoid areas as can be seen in Figure 4.

Treatment strategies and surgical intervention

Given the advanced nature of the tumor as indicated by the imaging findings, a high orchidectomy procedure was deemed necessary. This surgical intervention aimed for comprehensive resection of the tumor with wide margins, followed by adjuvant radiotherapy to improve local control and reduce the risk of distant metastasis. The collaborative efforts of the surgical and oncological teams, along with the inclusion of adjuvant therapies, aimed to optimize the treatment outcome and minimize the risk of recurrence.

Outcome and follow-up

**Figure 5:**
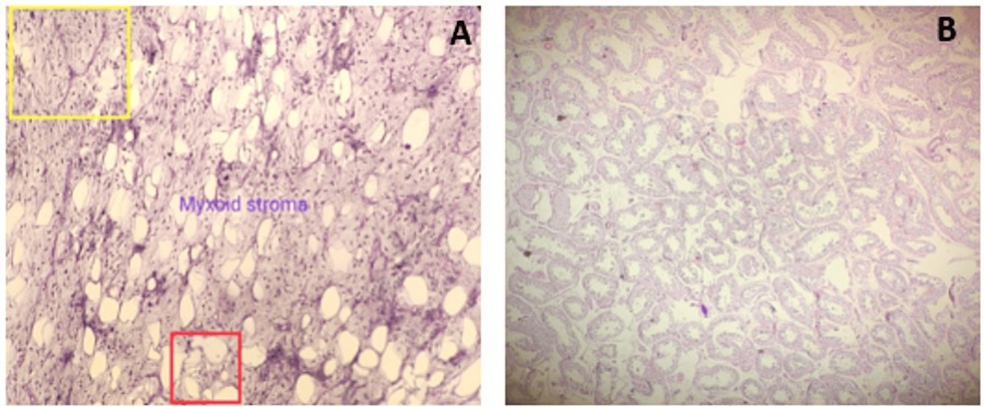
Microscopic examination of the mass revealed (A) monomorphic, stellate-shaped cells without atypia, chicken-wire type blood vessels (yellow square) (delicate thin-walled arborizing and curving capillaries), lipoblasts (red square), and myxoid matrix, (B) testicular tissue is composed of seminiferous tubules without tumor involvement.

The subsequent histopathological examination of the orchidectomy specimen uncovered monomorphic, stellate-shaped cells without atypia, chicken wire-type blood vessels, lipoblasts, and a myxoid matrix. These histopathological findings were crucial in confirming the diagnosis of a grade 2 right PTML (Figure 5A). Additionally, examination of the testicular tissue demonstrated the absence of tumor involvement in seminiferous tubules, as can be seen in Figure 5B, aiding in the comprehensive understanding of the lesion's nature.

## Discussion

PTML is an unusual clinical entity, hence presenting challenges in diagnosing, managing, and predicting patient outcomes. This case report presents a remarkable case of a 75-year-old male presenting with a giant PTML, expanding the breadth of documented cases and enriching the available literature on the topic. PTML is an exceptionally uncommon form of liposarcoma, representing only about 3% to 7% of sarcomas in the paratesticular region [3]. It arises from the adipose tissue in the paratesticular region and is distinguished by the presence of a myxoid matrix and scattered lipoblasts. This type of liposarcoma predominantly affects adults, with the typical age of presentation falling between 50 and 60 years of age [3]. Clinically, patients typically present with a painless, progressively enlarging mass in the paratesticular area, as observed in this case. However, due to the nonspecific symptoms, other benign and malignant paratesticular masses, such as lipomas, fibrous pseudo-tumors, and leiomyosarcomas, were included in the differential diagnosis.

In our case, imaging techniques identified a heteroechoic mass in the scrotum on USG, suggesting a solid tumor component. CECT revealed further characteristics consistent with PTML. An orchidectomy followed by a histopathological examination was performed on the mass and confirmed a grade 2 right PTML. The specimen's large size and involvement of the attached cord structure highlight the locally advanced nature of the tumor. Histologically, myxoid liposarcomas are characterized by a myxoid matrix with scattered lipoblasts, which were likely observed in the histopathological examination. The grade assigned to the tumor corresponds to its microscopic appearance and cellular atypia, which aids in determining its aggressiveness and potential for local recurrence or distant metastasis.

In comparison to dedifferentiated or well-differentiated liposarcoma, myxoid liposarcoma exhibits relative resistance to chemotherapy. The response rate of dedifferentiated liposarcoma to first-line chemotherapy, including doxorubicin alone (8%) or in combination with ifosfamide (17%), was notably lower, reaching only 25% [4]. The primary treatment for PTML involves surgical resection with wide margins, as achieved in this case through the high orchidectomy procedure [5]. Due to the locally infiltrative nature of the tumor and its potential for recurrence and metastasis, close surveillance and long-term follow-up with regular clinical assessments and imaging studies are essential.

In this case, the patient received adjuvant radiotherapy as part of a multidisciplinary treatment approach to improve local control and reduce the risk of distant metastasis. However, the effectiveness of adjuvant therapies in PTML is still under investigation, given the scarcity of reported cases and limited clinical data. The prognosis of PTML is influenced by various factors, including tumor size, histological grade, the extent of surgical resection, and the presence of metastasis. Giant tumors, like the one in this case, often exhibit more aggressive behavior and carry a higher risk of recurrence and metastasis. In more than half of the cases, recurrence is observed in paratesticular well-differentiated tumors, occasionally occurring at a later stage and progressing to low or high-grade dedifferentiation in some instances [6]. However, precise prognosis and long-term outcomes for PTML patients are difficult to determine due to the limited available data.

## Conclusions

Giant paratesticular liposarcomas remain an infrequent clinical finding. Given the absence of a universally accepted diagnostic and treatment protocol for these tumors, it is pivotal to opt for a high radical orchidectomy, to ensure comprehensive tumor removal, combined with consistent long-term monitoring due to the pronounced risk of recurrent tumor growth. Diagnostic tools such as ultrasound, CT scans, and MRI provide initial insights, yet the conclusive diagnosis stems from histological, immunohistochemical, and genetic evaluations. In situations where such tumors are suspected, immediate high inguinal orchidectomy combined with localized tumor resection is advised.
